# TRAIL-based gene delivery and therapeutic strategies

**DOI:** 10.1038/s41401-019-0287-8

**Published:** 2019-08-23

**Authors:** Hui-hai Zhong, Hui-yuan Wang, Jian Li, Yong-zhuo Huang

**Affiliations:** 1 Shanghai University College of Sciences, Shanghai, 200444 China; 2 State Key Laboratory of Drug Research, Shanghai Institute of Materia Medica, Chinese Academy of Sciences, Shanghai, 201203 China

**Keywords:** TRAIL, gene delivery, gene therapy, DNA, drug delivery systems, non-viral vectors, cancer therapy

## Abstract

TRAIL (tumor necrosis factor-related apoptosis-inducing ligand), also known as APO2L, belongs to the tumor necrosis factor family. By binding to the death receptor 4 (DR4) or DR5, TRAIL induces apoptosis of tumor cells without causing side toxicity in normal tissues. In recent years TRAIL-based therapy has attracted great attention for its promise of serving as a cancer drug candidate. However, the treatment efficacy of TRAIL protein was under expectation in the clinical trials because of the short half-life and the resistance of cancer cells. TRAIL gene transfection can produce a “bystander effect” of tumor cell killing and provide a potential solution to TRAIL-based cancer therapy. In this review we focus on TRAIL gene therapy and various design strategies of TRAIL DNA delivery including non-viral vectors and cell-based TRAIL therapy. In order to sensitize the tumor cells to TRAIL-induced apoptosis, combination therapy of TRAIL DNA with other drugs by the codelivery methods for yielding a synergistic antitumor efficacy is summarized. The opportunities and challenges of TRAIL-based gene delivery and therapy are discussed.

## Introduction

Nucleic acid-based therapy has been considered one of the most promising strategies for the treatment of various diseases [1]. Tumor necrosis factor (TNF) plays an important role in the homeostatic regulation of the immune system [2]. Although TNF is potent in causing tumor necrosis, the first two clinical trials of TNF-like molecules for cancer therapy failed because of lethal inflammatory shock syndrome and fulminant liver toxicity [3, 4]. Subsequentlyx, a novel TNF family member, TNF-related apoptosis-inducing ligand (TRAIL), was found [5, 6]; this protein is a type II transmembrane protein and can be released from the cell surface in soluble form via proteolysis [7]. Soluble TRAIL is nontoxic to normal cells, and in fact, there is a trace amount of endogenous TRAIL (~100 pg/mL) in healthy adult plasma [8, 9]. The TRAIL protein is expressed in various tissues—predominantly in the spleen, lung, and prostate—and on the surface of cytotoxic T cells and natural killer (NK) cells [10]. Its death receptors (DRs), DR4 and DR5, are overexpressed in many types of cancer cells. Importantly, TRAIL is capable of killing tumor cells without causing lethal adverse effects [11, 12].

**Fig. 1:**
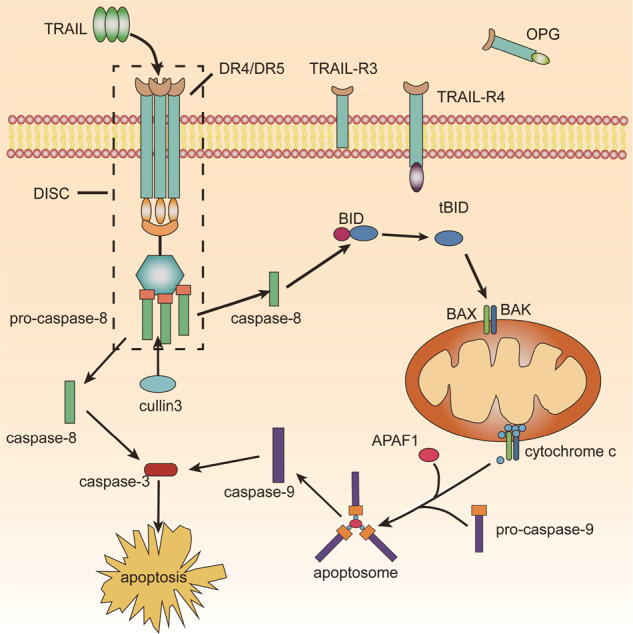
Pathway of TRAIL-induced apoptosis. TRAIL binds to five receptors, including four membrane-bound receptors (i.e., DR4, DR5, TRAIL-R3, and TRAIL-R4) and one soluble receptor (OPG). Only binding to DR4 or DR5 results in receptor trimerization and recruitment of FADD via the DDs of DR4 or DR5. After further recruitment of caspase-8, these proteins form a complex named DISC, which can activate caspase-8. In type I cells, caspase-8 activates caspase-3 and triggers apoptosis via the extrinsic pathway. However, in type II cells, the intrinsic pathway is triggered via caspase-8/BID/tBID, and consequently, BAX/BAK on the mitochondrial membrane is activated to induce the release of cytochrome *c*, which promotes the formation of the apoptosome with APAF1 and procaspase-9. Subsequently, activation of caspase-9 and caspase-3 is induced. Figure adapted from Fig. 2 in Ref. [7]

Apoptosis is an essential function of the maintenance of cellular homeostasis and prevents a number of diseases, including cancer [13]. Tumorigenesis is associated with defects in apoptosis regulation [14]. There are two major apoptotic pathways: the intrinsic, or mitochondrial, pathway usually induced by chemotherapy [10], and the extrinsic, or DR, pathway that mediates extrinsic programs of cell death, such as TRAIL-induced apoptosis. However, these two pathways usually associate with each other downstream via “crosstalk” [15]. The extrinsic pathway is activated by extracellular proapoptotic stimulators that bind to cell surface receptors [16]. There are five homologous human receptors for TRAIL: the full-length intracellular death domain (DD)-containing receptors DR4 [17] and DR5 (TRAIL-R2) [18–20]; the decoy receptor 1 (DcR1 or TRAIL-R3), which lacks an intracellular domain; [18, 19, 21] DcR2 (TRAIL-R4), which contains a truncated DD; [22, 23] and the soluble receptor osteoprotegerin (OPG). Among these receptors, only the binding of TRAIL to DR4 or DR5—because of their integrated intracellular structure—can induce an apoptotic effect; DR5 has the highest affinity for TRAIL [24]. However, TRAIL/DcR1 binding cannot induce downstream signaling, because DcR1 lacks an intracellular domain, while DcR2 and OPG act as NF-κB ligand receptors, which induce NF-κB activation but not apoptosis. After DR4 or DR5 binding to trimeric TRAIL, the intracellular DD structure of the DRs is altered, and binding to Fas-associated death domain-containing protein (FADD) then occurs. Then, FADD binds to procaspase-8/-10 via the death effector domain (DED) on the N-terminus, thereby forming DR4/DR5-FADD-procaspase-8/-10, which is called the death-inducing signaling complex (DISC). Oligomerization and autocatalysis of procaspase-8 leads to the activation of caspase-8, which consequently triggers cleavage of the effector caspases-3/-7/-9 to induce apoptosis. Furthermore, caspase-8 promotes the release of cytochrome *c*, inducing intrinsic apoptosis via the mitochondrial pathway in type II cells [25]. The TRAIL-induced apoptosis process is summarized in Fig 1.

The ability of the tumor-specific action of TRAIL to induce the apoptosis of cancer cells while sparing normal cells is attractive and renders TRAIL signaling a potential therapeutic target. To date, clinical trials of TRAIL have focused on recombinant TRAIL protein and antibodies against TRAIL-R (Table 1). However, clinical trials have shown inadequate treatment outcomes [26]. The recombinant form of TRAIL and anti-TRAIL-R antibodies, as well as their combination with other components, have not achieved the expected efficacy [7]. For example, the recombinant form of TRAIL did not exhibit significant antitumor effects, partially due to its short half-life [26, 27], while TAS266, an antibody targeting DR5, showed acute toxicity in a phase I clinical study [28].

**Table 1:** Clinical trials of TRAIL-based therapy

Formulation	Combination treatment	Disease	Clinical trial identifier
Mapatumumab (fully monoclonal antibody to TRAIL-R1)	Sorafenib	Advanced hepatocellular carcinoma	NCT01258608
	Sorafenib	Advanced hepatocellular carcinoma	NCT00712855
	Bortezomib	Relapsed or refractory multiple myeloma	NCT00315757
TRM-1 or HGS-ETR1 (monoclonal antibody to TRAIL-R1)	None	Relapsed or refractory non-Hodgkin’s lymphoma	NCT00094848
HGS-ETR2 (fully monoclonal antibody to TRAIL-R2)	Interferon gamma 1b	Solid tumors	NCT00428272
Conatumumab (fully monoclonal antibody to TRAIL-R2)	FOLFOX6 or Ganitumab or Bevacizumab	Advanced solid tumors	NCT01327612
Tigatuzumab (fully monoclonal antibody to TRAIL-R2)	Abraxane	Metastatic triple-negative breast cancer	NCT01307891
DS-8273a (antibody to TRAIL-R2)	Nivolumab	Advanced colorectal cancer	NCT02991196
	Nivolumab	Unresectable stage III or stage IV melanoma	NCT02983006
AMG 655 (fully monoclonal antibody to TRAIL-R2)	AMG 479 (fully human monoclonal antibody to IGF-1R) or Gemcitabine	Metastatic pancreatic cancer	NCT00630552
	AMG 479	Solid tumors	NCT00819169
	Doxorubicin	Unresectable soft tissue sarcoma	NCT00626704
	Vorinostat or Bortezomib	Relapsed or refractory low-grade lymphoma	NCT00791011
AMG 951 (recombinant human TRAIL)	Bevacizumab, Carboplatin, Paclitaxel	NSCLC	NCT00508625
Dulanermin (recombinant human TRAIL)	Placebo	NSCLC stage IV	NCT03083743
SCB-313 (recombinant human TRAIL-trimer fusion protein)	None	Malignant pleural effusions	NCT03869697
MSCTRAIL	Placebo	Adenocarcinoma of lung	NCT03298763

The clinical trials can be found at *https://www.clinicaltrials.gov*

There are three major limitations of TRAIL-based therapy: its short in vivo half-life [29, 30], its poor tumor-targeting efficacy, and resistance to TRAIL monotherapy [31, 32]. The emergence of nanotechnology has provided a useful tool to address these problems. TRAIL-based nanotherapies offer the potential to improve the stability of TRAIL and prolong its half-life in the bloodstream, to specifically deliver TRAIL to target sites and to overcome resistance to TRAIL [33]. Compared with direct administration of TRAIL proteins, TRAIL gene therapy also has the unique advantages of delivering TRAIL-encoding DNA into tumor cells to locally secrete the TRAIL protein on the membrane or into the tumor microenvironment, thereby overcoming the limitations of recombinant TRAIL protein. Notably, combination therapies of TRAIL with other anticancer agents via a codelivery system may solve the problem of TRAIL resistance.

TRAIL gene therapy also benefits from the “bystander” effect, by which not only the host cancer cells but also the neighboring cancer cells can be killed by both secreted and membrane-bound TRAIL [34]. TRAIL shows a unique advantage over other cell death-inducing ligands (e.g., Fas ligand, FasL), of which only the membrane-bound form can induce apoptosis, while the intrinsic soluble form cannot [35]. Liposome-bound TRAIL induces even more efficient apoptosis than the soluble form [36]. TRAIL-based gene therapy has been investigated in various types of tumors, such as hepatocellular carcinoma and cervical cancer.

Since the completion of the Human Genome Project, the development of gene therapy has accelerated. Gene therapy depends on the success of delivering specific nucleic acids to target sites by overcoming a series of biobarriers; in other words, it relies on the efficient delivery of vectors. Viral vectors are well known for their high transfection efficiency, which mainly relies on their ability to integrate genes into the genome of host cells. This approach increases the risk of insertion mutations at the integration site, especially if there are hotspots near prooncogenes. Thus, there is an urgent need to find a highly efficient vector with low genotoxicity and immunogenicity.

Nonviral vectors delivering genes typically via a nanosized platform are another option, with obvious advantages of safety and the high packing capacity of nucleic acids. In addition, advances in nanotechnology have provided good insight into rational designs for targeted delivery. Although numerous nonviral vectors have been developed, the amount of data from clinical trials has been very limited due to their low transfection efficiency [37]. Thus, there is a pressing need to develop nonviral vectors with increased efficiency. Nonviral vectors include cationic lipids, cationic polymers, and inorganic nanoparticles. Polyethyleneimine (PEI), for example, has high transfection efficiency in various cell lines. For the systemic delivery of gene therapeutics, delivery systems must cross a series of barriers, which is an important consideration in the design of delivery systems.

Regarding TRAIL-based therapy, viral vectors are prominently used for cell therapy, while nonviral vectors deliver plasmid-encoded TRAIL (pTRAIL) to targets. In this review, we summarize TRAIL-based gene therapeutic strategies and discuss the challenges facing clinical trials of TRAIL.

## TRAIL-based gene monotherapy

### Viral vectors

Most gene therapy clinical trials involved viral vectors [38]. Modified viruses such as retroviruses, lentiviruses, adenoviruses, and adeno-associated viruses (AAVs) are commonly used to deliver genes. Viruses are able to transfer genes into many cell types, with highly efficient transfection. Griffith et al. constructed a TRAIL-encoded adenovirus and found that rapid expression of the TRAIL protein and apoptosis of tumor cells were triggered by the activation of caspase-8 [39]. Oncolytic adenoviruses (OAds) can selectively replicate in cancer cells while sparing normal cells; thus, these viruses have been used in clinical trials for anticancer therapy. El-Shemi et al. applied systemic therapy with Ad-ΔB/ING4 (inhibitor of growth 4) plus Ad-ΔB/TRAIL in an orthotopic human hepatocellular carcinoma (HCC) -bearing mouse model. This study found that the combination of these agents elicited potent eradicative effects by inducing apoptosis and immune responses, and suppressing tumor angiogenesis without causing obvious overlapping toxicity [40].

In addition, the use of viral vectors has been explored for TRAIL-based cell therapy, which will be introduced in section 3.

### Nonviral vectors

Because of the inherent shortcomings of viral vectors, including their limited DNA packaging capacity, complicated production processes, broad tropism, cytotoxicity, immunogenicity, and tumorigenicity, nonviral vectors have also been widely investigated as an option for gene therapy [41]. In contrast to viral vectors, nonviral vectors have the advantages of low immunogenicity, high delivery capacity, and easy preparation [42, 43].

Before they reach the target cells, delivery systems need to cross a series of biobarriers. In the physiological environment, positively charged complexes are more prone to bind serum proteins and aggregate and thus be cleared rapidly [44, 45]. Shielding the positive charge using polyethylene glycol (PEG) or anionic materials such as γ-PGA can be helpful. Another challenge is selective accumulation at the target tissue, which requires specific designs, such as arming targeted ligands. Subsequently, the DNA of interest needs to be delivered to the nucleus for transcription [41]. Thus, the design of gene delivery systems should account for these considerations.

#### Polymers

Cationic polymers are an important class of nonviral vectors. Poly(*L*-lysine) (PLL) and polyethyleneimine (PEI) were developed for gene delivery in the 1990s. Subsequently, numerous cationic polymers have been developed and used, including poly[(2-dimethylamino) ethyl methacrylate] (pDMAEMA), poly(β-amino ester)s, and various carbohydrate-based polymers and dendrimers. PEI and its derivatives are the most commonly used polymeric vectors for gene delivery, with the advantage of the “proton sponge” effect, which facilitates the endosomal escape of gene drugs [46].

However, the major hurdles to overcome for the in vivo use of PEI are the substantial cytotoxicity related to its strong positive charge and the issue of its stability in the bloodstream [47]. To address these issues, modification of PEI and the surface coating have been investigated. For example, a γ-PGA corona can shield the positive charge of the PEI/DNA complex, thereby decreasing its toxicity and increasing its stability. It has been reported that the γ-PGA-coated branched PEI/pTRAIL complex can efficiently transfect pancreatic stellate cells expressing fibroblast growth factor receptors [48].

**Fig. 2:**
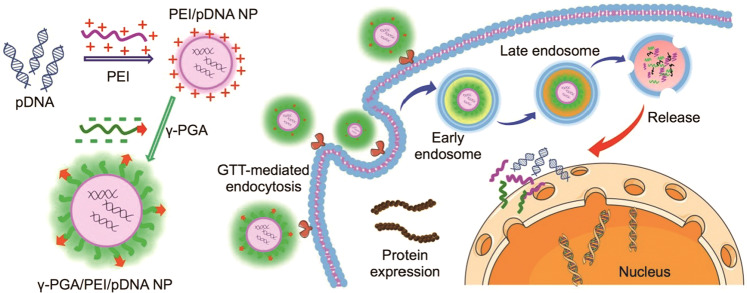
Schematic of the poly-γ-glutamic acid-based GGT-targeting and surface camouflage strategy for cervical cancer gene therapy. γ-PGA protects the positively charged complex from serum proteins and promotes GGT-mediated uptake. Reprinted with permission from [51]

Although PEI_25K_ is the gold standard for polymer transfection, its high molecular weight generally causes high toxicity. Therefore, many studies focusing on low-molecular-weight PEI have been reported. For instance, a gene delivery system for brain targeting was established by using PEI_10K_ modified with myristic acid (MC); MC-PEI_10K_/DNA nanoparticles could interact with the cell membrane via the hydrophobic segment on MC that can be incorporated into the phospholipid bilayer. MC-PEI_10K_/pORF-hTRAIL nanoparticles were effective against the growth of intracranial tumors [49]. Furthermore, cell-penetrating peptide (CPP)-modified and mannosylated low-molecular-weight PEI (termed Man-PEI_5k_–CPP) was constructed as a vector to deliver pTRAIL for colorectal cancer treatment. Man-PEI_5k_–CPP increased the cellular uptake efficiency and improved the efficiency of transfection [50]. Then, a ternary complex system was developed by a γ-PGA-based γ-glutamyl transpeptidase (GGT)-targeting and surface camouflage strategy via a layer-by-layer self-assembly method. Biodegradable polyanionic γ-PGA protected the PEI/pDNA complexes from interaction with body fluid components and interacted with tumor-overexpressed GGT, which can mediate the endocytosis of nanoparticles for cervical cancer gene therapy (Fig. 2) [51].

Hyaluronic acid (HA) also acts as a biocompatible polyanionic biomaterial for shielding positive charges. An HA-decorated polyethyleneimine-poly(*D, L*-lactide-co-glycolide) nanoparticle (PEI-PLGA NP) system was established for targeted codelivery of pTRAIL and gambogic acid (GA) for triple-negative breast cancer (TNBC) therapy [52]. GA was encapsulated into the hydrophobic core of PEI-PLGA NPs, while pTRAIL was adsorbed onto the positively charged NP surface. The HA coating on the PEI-PLGA NPs not only functioned as a shell to neutralize the excess positive charge on the NPs but also served as a targeting ligand by binding to the CD44 receptor on TNBC cells.

A series of terpolymers were synthesized via the enzyme-catalyzed copolymerization of lactone with dialkyl diester and aminodiol. Targeted delivery of pTRAIL to the tumor by the terpolymers resulted in significant inhibition of tumor growth with minimal toxicity in vivo, and the transfection efficiency was associated with the high molecular weight and increased hydrophobicity [53].

**Fig. 3:**
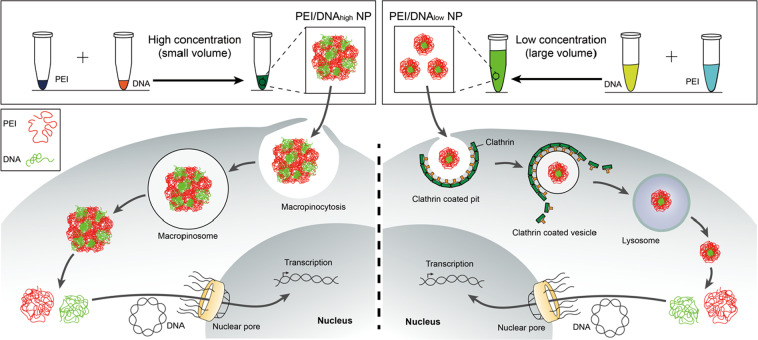
Transfection of the two groups of PEI/DNA NPs. Reprinted with permission from [54]

Intriguingly, it was found that preparation via a high concentration process (i.e., a small reaction volume) resulted in large PEI/DNA complexes that had a higher gene transfection efficiency than their small counterparts prepared at a low concentration (Fig. 3) [54]. The mechanisms were associated with macropinocytosis and fast dissociation. Accordingly, large-sized PEI/pTRAIL complexes exhibited increased anticancer efficacy via local or regional administration in subcutaneous xenograft and peritoneal xenograft mouse models.

**Fig. 4:**
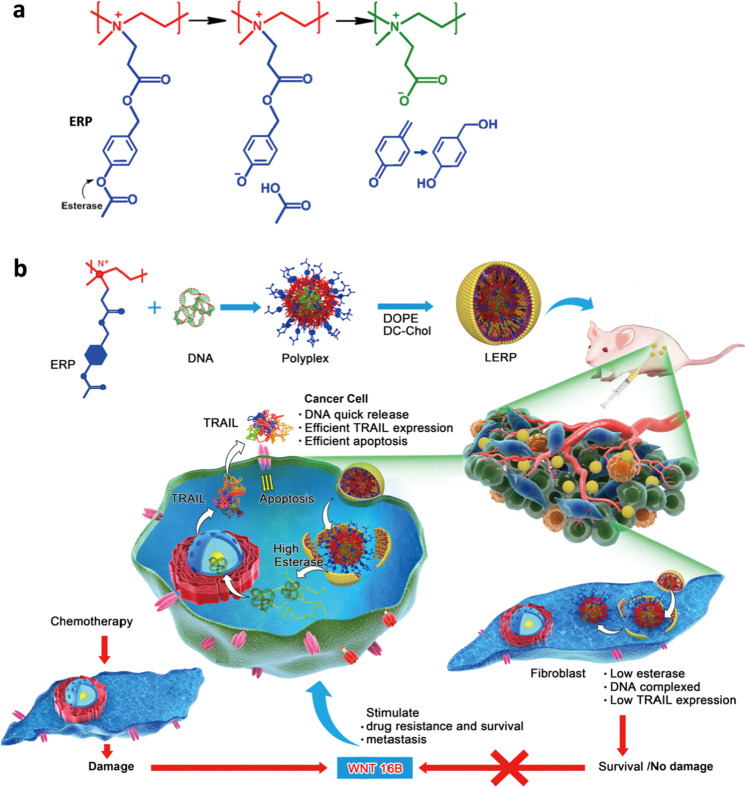
Illustration of the esterase-responsive charge‐reversal polymer (ERP) and its lipid‐coated esterase-responsive polyplexes with TRAIL plasmid for cancer gene therapy. **a** The ERP is a PEI whose amines are quaternized with propionic 4‐acetoxybenzyl ester. Hydrolysis of the phenolic acetate triggers the elimination of p-hydroxymethylphenol and consequent conversion of the cationic polymer into a zwitterionic form. **b** ERP condenses plasmid DNA into the polyplexes, which are easily coated with DC-Chol/DOPE lipids to form lipidic esterase‐responsive polyplexes (LERPs). After i.p. injection into nude mice bearing HeLa cell-derived tumors, tumor cells internalize LERPs into the cytosol, which is rich in esterases. The LERPs disassemble and release the polyplexes to allow the esterases to trigger the charge reversal of the ERP and thus plasmid release. These free plasmids enter the nucleus for effective gene expression, inducing apoptosis when delivering the TRAIL gene. In tumor fibroblasts, the low esterase level cannot efficiently induce the charge reversal process, and the TRAIL plasmids will not be expressed, preventing WNT16B production. Reprinted with permission from [56]

Poly(beta-amino ester)s (PBAEs) are a class of cationic synthetic polymers that can be used as nonviral gene carriers. These compounds are easy to synthesize, bind effectively to DNA, and are hydrolytically degradable under physiological conditions. Tzeng et al. reported the use of PBAE nanoparticles containing pTRAIL for the selective transfection of cancer cells and the induction of apoptosis in several human cancer cells [55]. Additionally, Shen et al. developed an ingenious vector comprising quaternary amines carrying *N*-propionic 4-acetoxybenzyl ester substituents; the 4-acetoxybenzyl ester group can undergo rapid intracellular esterase-catalyzed hydrolysis, which subsequently triggers a reversal of the polymer’s charge from cationic to zwitterionic [56]. Due to the high cytosolic esterase activity in cancer cells but low activity in fibroblasts, the loaded pTRAIL can be delivered specifically into cancer cells without damaging fibroblasts, preventing the expression of WNT16B (Fig. 4) [56]. The same group also synthesized another enzyme-responsive cationic polymer, poly(PQDEA), which is rapidly hydrolyzed by intracellular esterases to form anionic poly(acrylic acid) to confer low cytotoxicity and fast release of DNA [57]. PQDEA/DNA polyplexes were further coated with a lipid layer to generate serum-stable lipidic polyplexes (LPQDEA/DNAs) for in vivo use. LPQDEA/pTRAIL strongly inhibited tumor formation as effectively as paclitaxel but gave less tumor relapse and longer survival.

In addition, some inorganic materials have been modified with polymers for pTRAIL delivery. For example, a sandwich-type PEI-coated gold nanocomposite coloaded with pTRAIL and nuclear-targeted dexamethasone (Dexa) (termed Au-PEI/pTRAIL/PEI-Dexa) exhibited efficient transfection and significantly inhibited the growth of Hep3B tumors [58]. A targeted iron oxide NP coated with a chitosan-PEG-PEI copolymer and chlorotoxin was developed; this NP efficiently delivered pTRAIL into human T98G GBM cells and induced the secretion of TRAIL [59]. Further, systemic administration to mice bearing T98G-derived flank xenografts resulted in almost imperceptible tumor growth and induced apoptosis in tumor tissue. To overcome treatment limitations of adenoid cystic carcinoma, the Fe_3_O_4_-PEI-plasmid complex (FPP) was generated, in which iron oxide NPs were modified by positively charged PEI to enable them to carry pACTERT-TRAIL [60]. The efficiency of FPP-mediated transfection was sixfold higher than that of PEI alone or of Lipo2000, and FPP-mediated TRAIL gene transfer efficiently inhibited SACC-83 tumor growth.

#### Dendrimers

Dendrimers, which are characterized by their well-defined size and low polydispersity index, have been widely used for drug and gene delivery [61]. Jiang et al. reported a gene vector generated by polyamidoamine (PAMAM) and Angiopep-2 conjugation via bifunctional PEG (PAMAM-PEG-Angiopep) [62]. The PAMAM-PEG-Angiopep/pORF-TRAIL NPs could penetrate the blood–brain barrier (BBB) and target glial cells based on Angiopep-2-mediated delivery, with hight transfection efficiency. Another strategy has been reported in which transferrin (Tf) was conjugated to a generation 3 diaminobutyric polypropylenimine (DAB) dendrimer; this delivery system harbors plasmids encoding for TNF-α, TRAIL, or IL-12 and leads to therapeutic effects on prostate tumors following intravenous administration [63]. In addition, lactoferrin (Lf)-bearing DAB dendriplexes showed similar efficacy [64].

Later, coumarin-anchored low-generation dendrimers (G1 PAMAM dendrimers) were established to improve DNA binding and gene delivery [65]. The coumarin moieties endowed these materials with light-responsive drug delivery behaviors, and the drug-loaded nanoparticles exhibited complementary anticancer activity through the codelivery of 5-fluorouracil and pTRAIL. A triazine-modified dendrimer, G5-DAT66, was synthesized and used as a vector for pTRAIL therapy, showing higher transfection efficacy than commercial transfection reagents such as Lipo2000 and SuperFect [66]. Furthermore, in vivo studies demonstrated that G5-DAT66/pTRAIL efficiently inhibited tumor growth in osteosarcoma-bearing mice. Pishavar et al. synthesized a vector composed of PAMAM dendrimers modified with alkyl-carboxylate chains, PEG, and cholesteryl chloroformate for delivering pTRAIL, and these PAMAM G4-alkyl-PEG (3%)-Chol (5%)-TRAIL complexes inhibited CT26 tumor growth in mice [67].

#### Peptides and proteins

Cationic peptides rich in residues such as lysines or arginines are able to condense DNA. Furthermore, conjugation of peptide ligands to delivery systems allows targeting to specific types of cancer cells [68]. A biomimetic vector was developed for delivering pTRAIL to tumor; it contained an adenovirus μ peptide for pDNA condensation, a synthetic cyclic peptide for tumor-targeting and intracellular delivery, a pH-responsive synthetic fusogenic peptide for endosome escape, and a nuclear localization signal from human immuno-deficiency virus for intranuclear delivery [69]. Up to 62% of ZR-75-1 breast cancer cells were killed after exposure to pTRAIL in complexation with this vector. A dimerized HIV-1 TAT peptide was used to formulate a nanoparticle vector (dTAT NP) to leverage the efficiency of this cell penetration strategy for tumor-targeted gene delivery [70]. In cell culture, dTAT NP was an effective pDNA transfection vector with negligible cytotoxicity. Gene expression in tumor tissues lasted for >14 days after intratracheal administration. Bolus administration of dTAT NP–encapsulated pTRAIL markedly attenuated the growth of tumors derived from Lewis lung carcinoma cells. Phosphatase and tensin homolog (PTEN) and TRAIL genes loaded into zein nanoparticles showed antiproliferative activity against HepG2 cell lines, indicating their potential for gene therapy for the treatment of HCC [71].

#### Liposomes

Cationic lipids have been widely used for nucleic acid delivery and for advances in gene delivery through their molecular design. Cationic lipids can spontaneously form specific types of complexes for condensing and encapsulating DNA into particles [72].

Huang et al. reported a novel lipid (1,2-di-(9Z-octadecenoyl)-3-biguanide-propane (DOBP)) that was elaborately designed by utilizing biguanide as the cationic head group [73]. This novel cationic lipid acted as a gene carrier and had a metformin-like antitumor activity via activation of the AMPK and inhibiting the mTOR pathways. DOBP-LPDTRAIL NPs showed potent efficacy against tumor progression. Then, these NPs were used to transfer a secreted form of TRAIL (sTRAIL) to tumor-associated fibroblasts in order to secrete cytotoxic proteins to tumor cells via lipid-coated protamine [74]. sTRAIL triggered apoptosis in tumor cell nests adjacent to TAFs. Furthermore, sTRAIL converted the residual fibroblasts to a quiescent state, thus arresting the tumor growth and remodeling the microenvironment to facilitate the second-wave nanotherapy. The system showed good efficacy in an orthotopic xenograft model of human pancreatic cancer, where the desmoplastic stroma is a major barrier to the delivery of therapeutic nanoparticles.

**Fig. 5:**
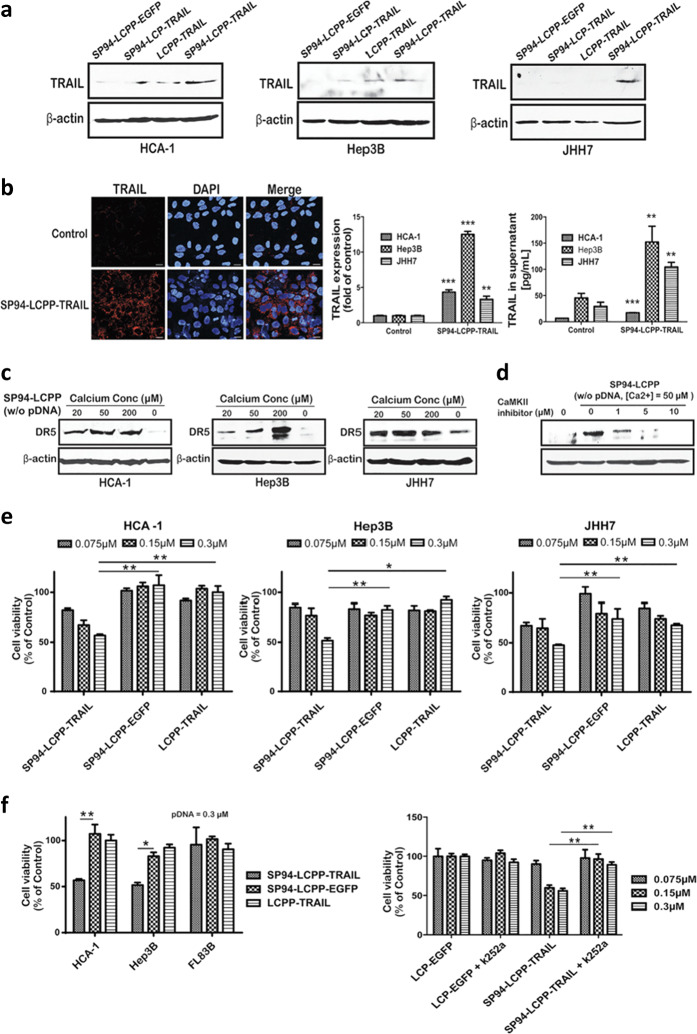
TRAIL expression and TRAIL-induced cytotoxicity in HCC cells by SP94-targeted LCPP NPs in vitro. **a** SP94-targeted LCPP NPs increased TRAIL expression in both human and murine HCC cells, as assessed by Western blotting. **b** TRAIL expression and secretion were quantified by confocal microscopy and ELISA, respectively. **c** SP94-LCPP NPs without pDNA significantly increased DR5 expression in a dose-dependent manner in HCC cells. **d** The CaMKII inhibitor k252a prevented the effects of LCPP NPs on DR5 upregulation. **e**, **f** Treatment with TRAIL pDNA loaded into SP94-LCPP NPs showed higher cytotoxicity in HCC cells than to control cells. However, these NPs exhibited slight cytotoxicity in murine hepatocyte FL83B cells. Reprinted with permission from [75]

Chen et al. developed a tumor-targeted LCPP (lipid/calcium/phosphate/protamine) NP to deliver pTRAIL into HCC cells in a mouse model of HCC [75]. pTRAIL was entrapped in a pH-responsive calcium phosphate (CaP) core, and protamine was included to direct intranuclear delivery. TRAIL resistance could be reversed by intracellular release of Ca^2+^ from the CaP core that induced DR5 up‐regulation through CaMKII activation (Fig. 5).

#### Other vectors

Gong et al. developed a well-tailored and versatile “core-shell” ternary system (RRPHC) for systemic gene delivery to treat aggressive melanoma [76]. This system consisted of a core of fluorinated polymers (PFs) that bound to a plasmid (pDNA) and a negatively charged multifunctional RRPH (RGD-R8-PEG-HA) shell constructed by grafting a hyaluronan polymer with PEG side chains, which were further conjugated with the R8-RGD tandem peptide on the distal side, simultaneously targeting the CD44 receptors and integrin α_V_β_3_ receptors overexpressed on the neovasculature and most malignant tumor cells. Systemic injection of the proapoptotic mTRAIL plasmid by RRPHC ternary complexes inhibited the melanoma growth, without noticeable side toxicity. Next, this group reported a similar system of an artificial virus core-shell to target cancer stem cell-like cells [77]. The intravenously injected nanoparticles accumulated at the tumor sites while reducing the exposure to the normal tissues, and efficiently arrest the tumor growth, without obvious systemic toxicity.

## TRAIL-based cell therapy

Mesenchymal stem cells (MSCs) are a population of fibroblast-like cells originally isolated from bone marrow and other tissues, including adipose tissue, peripheral blood, umbilical cord blood, and Wharton’s jelly, among others [78, 79]. MSCs have therapeutic potential in several pathological conditions and unique immunological features [80–82]. In recent years, the cellular vehicle function of MSCs has been used to transfer TRAIL to the tumor parenchyma [83–85].

Lee et al. reported a novel application of magnetic core-shell nanoparticles for the dual purpose of delivering and activating a heat-inducible gene vector that encodes TRAIL in adipose-derived mesenchymal stem cells (AD-MSCs) [86]. This group developed a plasmid harboring the heat shock protein 70B’ (HSP70B0) promoter. The magnetic core-shell nanoparticles (MC NPs) was composed of ZnFe^2^O^4^ magnetic nanoparticle core and mesoporous silica shell, as well as a surface coating of PEI for DNA binding. The MC NPs facilitated the intracellular delivery of the heat-inducible plasmid into AD-MSCs via magnetic guidance, and after systemic injection, the engineered AD-MSCs could home to tumors/metastases. TRAIL expression could be specifically activated via the induction of mild magnetic hyperthermia (~41 °C). This system enhanced control over the activation of stem cell-based gene therapies.

It was reported that human MSCs were transduced with TRAIL and the IRES-eGFP reporter gene under the control of the tetracycline promoter using a lentiviral vector [87]. The transduced and activated MSCs led to the apoptosis and death in various cancer cells in coculture experiments. The in vivo studies demonstrated that the i.v. injected TRAIL-expressing MSCs significantly arrested the tumor growth.

**Fig. 6:**
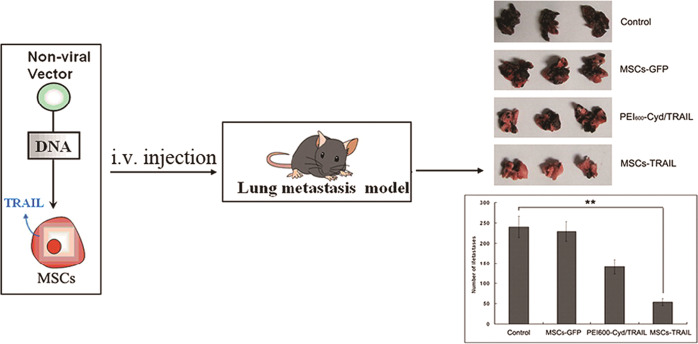
The nonviral vector PEI_600_-Cyd was used to transduce TRAIL plasmid into MSCs. TRAIL-armed MSCs showed a lung tumor-homing ability and an antitumor effect on lung metastasis-bearing C57BL/6 mice after i.v. injection. Reprinted with permission from [88]

Gao et al. transfected pTRAIL into MSCs with a nonviral vector, PEI_600_-Cyd, prepared by linking low-molecular-weight polyethyleneimine (PEI) and β-cyclodextrin (β-CD) [88]. The lung tumor homing ability of MSCs expressing TRAIL in vivo proved to be efficient for lung metastasis therapy. Fig. 6 shows a schematic of the specific process of this cell-based TRAIL therapy.

Human umbilical cord-derived mesenchymal stem cells (HUMSCs) were transfected by lentiviral vectors coding the sTRAIL with the alpha-fetoprotein (AFP) promoter, and the treatment efficacy of these engineered HUMSCs on orthotopically implanted hepatocarcinomas in mice was examined [89]. HUMSCs could migrate to the hepatocarcinoma, where the AFP promoter was triggered by the early hepatic differentiation of HUMSCs, and expressed sTRAIL at the cancer cells and yielded significant antitumor activity. Dominici et al. transduced human AD-MSCs with a retroviral vector encoding full-length human TRAIL [84]. AD-MSCs could target various cancer cell lines in vitro, and reverse the TRAIL resistance by coadministration of bortezomib. These AD-MSCs targeted to tumors and induced apoptosis, without apparent side toxicity. The engineered MSCs with TRAIL expression also induced apoptosis by cell-to-cell contact in the TRAIL-resistant Ewing sarcoma (EWS) that is insensitive to AMG655, an antibody against the death receptor DR5, too, and the treatment effect was confirmed in two orthotopic models of EWS [85]. Despite these encouraging results, there is some concern regarding the safety of inoculating both wild-type and genetically modified MSCs, especially regarding their possible damaging effects on normal organs, malignant transformation, and promotion of cancer growth [90]. Then, researchers incorporated suicide genes—the herpes simplex virus Thymidine Kinase (HSV-TK) and Cytosine Deaminase genes—into MSCs to control their fate once infused [91]. This approach is based on a variant of human caspase-9 that binds with high affinity to a synthetic, bioinert small molecule (AP20187), leading to cell death [92].

Conventionally, MSCs have been genetically modified for cancer therapy by using viral vectors that can elicit oncogenicity, thus limiting their use in clinical trials. Chen et al. used nonviral agents such as a polylysine-modified polyethyleneimine (PEI-PLL) copolymer to generate genetically engineered MSCs with suicide genes, namely, HSV-TK and TRAIL [93]. The MSCs armed with suicide genes along with prodrug ganciclovir can induce significant antitumor effect to glioblastoma by intratumoral injection both in vitro and in vivo. Another study on delivering the TRAIL gene for stem cell-mediated gene therapy was conducted by using nonviral vectors (a less efficient but safer method). Na et al. prepared the polyplexes of pTRAIL and bPEI, and photochemical internalization (PCI) was applied to improve the polyplex entrapping in hMSCs and enhance the transfection efficiency of pTRAIL; the tumor-homing hMSCs could also increase the TRAIL secretion in the tumors [94]. PCI-mediated polyplex loading significantly enhanced TRAIL expression in stem cells, and that homing ability enhanced cancer targeting. Exposure of polyplex-loaded hMSCs (pTRAIL/bPEI@hMSCs) to laser irradiation resulted in a beneficial therapeutic antitumor effect in a xenograft mouse model.

In addition to MSCs, Hu et al. reported a DC cell-based therapy for colon cancer cells [95]. Tyrosine kinase receptor 3 ligand (FL) and TRAIL plasmids were constructed for combination therapy. FL, a hemopoietic growth factor, is important in progenitor cell proliferation and differentiation, which can enhance the proliferative and antitumor effect of DCs. These two plasmids were transfected into DCs by Lipo2000. The combination of FL-carrying DCs and TRAIL-carrying DCs showed a good level of apoptosis in colon cancer [96].

## TRAIL-based combination therapy

Although TRAIL-induced apoptosis is an attractive therapeutic target, resistance to TRAIL readily develops during treatment. This therapeutic resistance derives from two sources: intrinsic resistance in some highly malignant tumors [97–99] and acquired resistance after repeated exposure to TRAIL [100]. Resistance to TRAIL is conferred by multiple receptors and involves a series of signaling pathways and activation of inhibitory molecules [99].

**Fig. 7:**
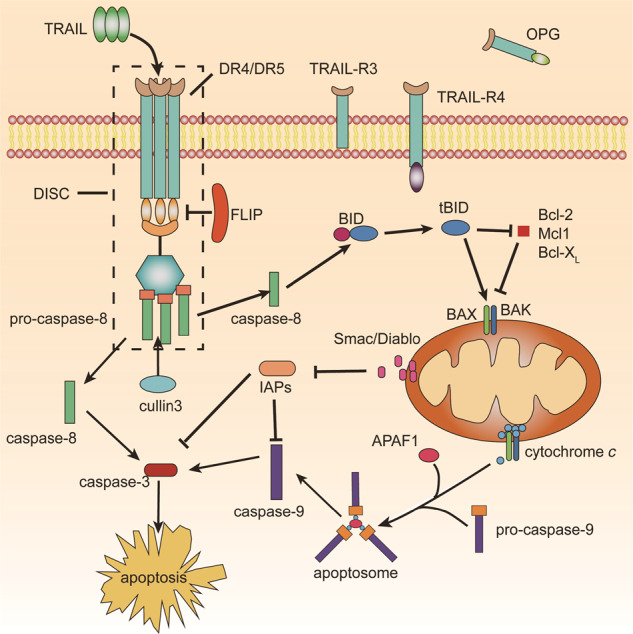
Inhibition of TRAIL signaling results in TRAIL resistance. When the DISC assembles, FLIP (FLICE-like inhibitory protein) can competitively bind to FADD and limit the recruitment of caspase-8. While Bcl-2 and Bcl-X_L_ can inhibit Bax and Bak, tBID plays roles in inhibiting Bcl-2 and Bcl-X_L_ and activating Bax and Bak in type II cells. IAPs can strongly inhibit the activation of effector caspases, as Smac/Diablo is needed to interact with IAPs to release effector caspases. Figure adapted from Fig. 2 in Ref. [7]

TRAIL signaling begins with the binding of TRAIL to the death receptors. First, binding to decoy receptors (e.g., TRAIL-R3 and R4) will not lead to the activation of downstream TRAIL-signaling. Second, mutation or downregulation of the functional DRs (e.g., DR4 or DR5) can also result in resistance. For instance, transfection of mutated DR4 into SW480 colon cancer cells caused a lower efficacy of cell killing than transfection of the wild-type counterpart [101]. Low expression of DR5 contributed to TRAIL resistance in anti-DR5 antibody therapy [102]. In this case, upregulation of DR5 is a therapeutic strategy for sensitization to TRAIL treatment [101]. Third, the multifunctionality of downstream TRAIL signaling also induces resistance. The assembly of the DISC can be inhibited by apoptosis inhibitors. For example, cFLIP, with a similar structure to caspase-8, can inhibit caspase-8 activation after binding to FADD [103]. The ratio of cFLIP/caspase-8 is correlated with TRAIL resistance in various tumors, such as hepatocellular carcinoma and Burkitt lymphoma [104, 105]. In type II cells, TRAIL-initiating apoptosis is mainly mediated by the mitochondrial pathway (Fig. 7). Upregulation of the proapoptotic proteins Bax and Bak promotes cell death, while the antiapoptotic proteins Bcl-2 and Bcl-X_L_ determine cell survival [106, 107]. Furthermore, inhibitors of apoptosis proteins (IAPs) can block apoptosis by inhibiting the activity of the effector caspases (e.g., caspase-3 and caspase-9), and overexpression of IAPs can confer TRAIL resistance [108, 109]. However, this effect can be reversed by IAP antagonists such as Smac/Diablo, which promotes apoptosis by interacting with IAPs [110, 111]. The mechanisms are summarized in Fig. 7.

In addition, epigenetic changes play a role in TRAIL resistance via the regulation of caspase-8 gene expression, and DNA methylation can restore apoptosis in TRAIL-resistant tumor cells. TRAIL resistance is also related to NF-κB and mitogen-activated protein (MAP) kinases. However, these signaling pathways showed both proapoptotic and antiapoptotic effects [112].

In conclusion, DR dysfunction and overexpression of cFLIP, Bcl-2, Bcl-X_L_, and IAPs contribute to TRAIL resistance, and therefore, regulation of DRs and inhibition of antiapoptotic proteins resensitizes cells to TRAIL treatment [113].

The resistance of cancer cells to TRAIL has encouraged the investigation of combination therapy. In recent years, many multifunctional drug delivery systems, including liposomes, micelles, and polymeric nanoparticles, have been developed for the codelivery of genes and drugs. Here, we summarize the delivery and therapeutic strategies of combinations of pTRAIL and different types of drugs.

### Combination with TRAIL sensitizers

**Fig. 8:**
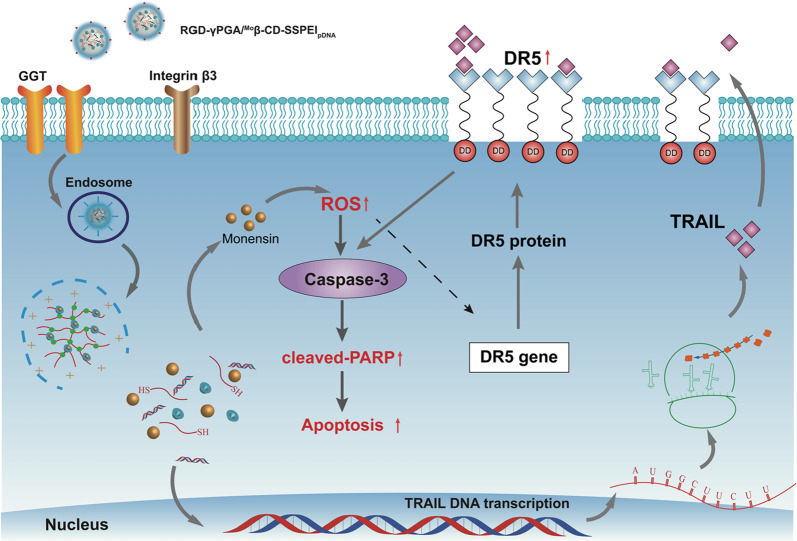
The process of codelivery of pTRAIL and monensin nanocomplexes. As a TRAIL sensitizer, monensin upregulates DR5 and sensitizes tumor cells to TRAIL. Reprinted with permission from [115]

The combination of TRAIL with its sensitizers is a promising strategy to overcome TRAIL resistance. It was found that polyether ionophore antibiotics (e.g., monensin) can overcome TRAIL resistance via endoplasmic reticulum stress induction, DR5 upregulation and cFLIP downregulation [114]. Huang et al. constructed a biocompatible nanosystem for the codelivery of pTRAIL and monensin, in which low-molecular-weight PEI_1.8k_ (LMW-PEI) was intermolecularly crosslinked via disulfide bonds using sulfhydryl β-cyclodextrin as a linker (Fig. 8) [115]. The resulting β-CD-SSPEI carrier, which can bind efficiently with pTRAIL, can serve as a carrier for codelivery, while monensin is encapsulated inside the cavities of the β-cyclodextrin molecules. The ^Mo^β-CD-SSPEI_pTRAIL_ nanocomplex can further be modified by a polyanionic polymer γ-PGA, thus protecting the nanocomplex from interaction with serum proteins and consequently extending its half-life in the bloodstream. Furthermore, γ-PGA/^Mo^β-CD-SSPEI_pTRAIL_ can achieve tumor targeting via specific binding between γ-PGA and tumor-overexpressed GGT, which can mediate the endocytosis of the nanocomplex. Inside cancer cells, the acidic endosomal environment facilitates the detachment of γ-PGA, whose conformation is pH-dependent, and the exposed PEI facilitates endosomal escape. Importantly, the intermolecular crosslinks of PEI can be degraded, and pTRAIL and monensin can be released. Monensin can increase intracellular ROS levels and induce apoptosis. Moreover, DR5 expression is upregulated, thus synergizing with TRAIL-based treatment for colon cancer gene therapy.

### Combination with chemotherapeutic drugs

TRAIL exhibits improved efficacy in combination with chemotherapy because chemotherapeutic agents can sensitize tumors to TRAIL-induced apoptosis via crosstalk between the intrinsic and extrinsic pathways of cell death [116]. The chemotherapeutic drugs doxorubicin (DOX) and paclitaxel (PTX) were explored to determine their synergistic antitumor effect with pTRAIL. Ebrahimian et al. reported a vector composed of polypropylenimine (PPI) modified with 10-bromodecanoic acid for the codelivery of pTRAIL and DOX for tumor therapy [117]. In addition, a host–guest conjugated nanoparticle for the codelivery of DOX and pTRAIL was designed [118]. The Adamantane-conjugated DOX (Ad-Dox, guest component) and the PEI-cyclodextrin conjugates (PEI-CD, host) were self-assembled into the supramolecular PEI-CD/Ad-Dox, which further bound with TRAIL DNA to form the PEI-CD/Ad- Dox/pDNA SNPs. The SNPs exhibited the enhanced therapeutic efficacy with the significantly increased survival rate of the tumor-bearing mice.

Jiang et al. investigated the codelivery of the hTRAIL-encoding plasmid open reading frame (pORF-hTRAIL) and DOX using a tumor-targeting carrier, a peptide HAIYPRH (T7)-conjugated polyethylene glycol-modified polyamidoamine dendrimer (PAMAM-PEG-T7) [119]. In this system, approximately 375 DOX molecules were bound to one pORF-hTRAIL molecule, and T7 served as a ligand targeting tumor cell-overexpressed transferrin receptors. This codelivery system induced apoptosis of tumor cells and efficiently inhibited Bel-7402 tumor growth in vivo. Furthermore, the combination therapy strategy was further applied to glioma, in which dendrigraft poly-*L*-lysine (DGL), modified by the T7 peptide and conjugated with DOX via a pH-sensitive hydrazone bond, was used to deliver pORF-hTRAIL to glioma tissue [120]. In addition, other dual targeting systems have also been developed for the codelivery of DOX and pORF-hTRAIL for glioma treatment [121, 122].

Regarding the combination of PTX and TRAIL, an angiopep-2 peptide-modified cationic liposome (ANG-CLP) for the efficient codelivery of pEGFP-hTRAIL and PTX to glioma was reported. Angiopep-2 can target the low-density lipoprotein receptor-related protein (LRP) overexpressed on the BBB and on glioma cells [123]. Lu et al. developed two kinds of nanoparticles for the delivery of pORF-hTRAIL and PTX to glioma tissues [124, 125]. pORF-hTRAIL was delivered by c(RGDyK)–poly(ethylene glycol)–polyethyleneimine (RGD–PEG–PEI). RGD can bind to integrin α_V_β_3,_ which is overexpressed in the neovasculature and on U87 glioblastoma cells. PTX was loaded in a CDX–poly(ethylene glycol)–block-poly(lactic acid) micelle. CDX is a peptide derived from the loop II region of the snake neurotoxin candoxin, with a high-binding affinity to nicotinic acetylcholine receptors (nAChRs). Dominici et al. investigated the combination of pTRAIL and PTX for MSC therapy [126]. PTX restored the sensitivity of pancreatic cancer to MSC-delivered TRAIL by reverting its prosurvival gene expression profile. Additionally, a combination of cisplatin and TRAIL with high anticancer activity was found [127].

These results indicate that TRAIL gene therapy in combination with chemotherapy can be promising for ovarian cancer therapy. Although it is not fully known how chemotherapy sensitizes cells to TRAIL-induced apoptosis, the effect may be related to a p53-independent pathway, in addition to changes in the expression of proteins involved in TRAIL signaling, which also play an important role [128].

### Combination with intracellular apoptosis-induced agents

As TRAIL induces extracellular apoptosis, its combination with agents mediating the intracellular apoptosis pathway can yield a synergistic effect. Histone deacetylase inhibitors (HADCi) induce cell cycle arrest and apoptosis in tumor cells, and HADCi can resensitize TRAIL-resistant cancer cells. Codelivery of pTRAIL and vorinostat (SAHA) with a reactive oxygen species (ROS)-triggered charge reversal polymer (B-PDEAEA) produced a good antitumor effect [129]. The increased transfection efficiency of pTRAIL and SAHA-induced ROS accumulation caused significant apoptosis of the cancer cells. The MSCs-based gene therapy for antiglioma was developed, in which hAT-MSCs was transfected by pIRES2-EGFP-sTRAIL [130]. The engineered MSCs effectively inhibited the proliferation of malignant glioma cells and upregulated DRs, yielding a potent antiglioma effect in combination with panobinostat.

IAPs play an important role in cancer cell resistance to TRAIL, and the combination of TRAIL with IAP antagonists can help sensitize cells to TRAIL-induced apoptosis. Ge et al. demonstrated that an oncolytic adenovirus coexpressing TRAIL and Smac, in combination with the cyclin-dependent kinase (CDK) inhibitor SNS-032, synergistically reinforced their individual anti-pancreatic cancer activities, and SNS-032 enhanced ZD55-TRAIL-IETD-Smac-induced apoptosis [131]. Shi et al. developed an AAV-mediated gene therapy that was characterized by coexpression of TRAIL with miR-221-Zip, which produced a synergistic effect on the enhanced apoptosis induction via the sensitizing effect of miR-221-Zip by upregulation of PTEN and downregulation of survivin [132].

Compared with TRAIL monotherapy, combination therapy provides enhanced treatment outcomes. Therefore, combinations including TRAIL-mediated therapy are a promising approach for cancer therapy.

## Perspective

TRAIL is a promising drug candidate for the treatment of many cancers. The investigation of TRAIL-based therapy in clinical trials focuses on recombinant TRAIL proteins and anti-TRAIL antibodies, but delivery and drug resistance issues are the main hurdles in the successful translation of these results. TRAIL-based gene therapy is another potential treatment approach. Importantly, the codelivery systems loaded with pTRAIL and drugs combined with TRAIL have been actively explored. The development of safe and efficient gene vectors is the central issue for gene therapy. The elegant design of advanced systems provides the tumor-targeted codelivery of both agents, thus achieving synergistic treatment effects. In addition, it is necessary to identify proper biomarkers for maximizing TRAIL-based therapy, which can also provide helpful information for the appropriate selection of drug combinations. However, clinical trials have made little progress. Efforts should be directed toward the rational design of appropriate formulations of TRAIL to improve its in vivo pharmacokinetic profile and the exploration of biomarkers specific to TRAIL-based therapy. The future application of TRAIL may benefit from a better understanding of the anticancer mechanisms of TRAIL as well as its combination with TRAIL sensitizers. It is expected that as precision medicine progresses, TRAIL-mediated antitumor functions will be better understood to allow the development of precise and effective TRAIL-based treatments for patients.
